# Reply to Teng et al. Comment on “Drozdowska-Szymczak et al. Incidence and Risk Factors of Cholestasis in Newborns with Hemolytic Disease—A Case-Control Study. *J. Clin. Med.* 2024, *13*, 3190”

**DOI:** 10.3390/jcm13237048

**Published:** 2024-11-22

**Authors:** Agnieszka Drozdowska-Szymczak, Natalia Mazanowska, Tomasz Pomianek, Artur Ludwin, Paweł Krajewski

**Affiliations:** 1 Department of Neonatology and Neonatal Intensive Care, Institute of Mother and Child, Kasprzaka 17a, 01-211 Warsaw, Poland; agnieszka.drozdowska@imid.med.pl (A.D.-S.); tomasz.pomianek@imid.med.pl (T.P.); pawel.krajewski@imid.med.pl (P.K.); 2 Department of Obstetrics and Gynecology, Institute of Mother and Child, Kasprzaka 17a, 01-211 Warsaw, Poland; 3 Department of Obstetrics and Gynecology, Medical University of Warsaw, Pl. Starynkiewicza 1/3, 02-015 Warsaw, Poland; ludwin@cm-uj.krakow.pl

We would like to express our sincere gratitude for your thoughtful review of our article [1] and for providing such valuable and constructive feedback [2]. Your insights are very important to us, and we appreciate the time and effort you have dedicated to evaluating our work.

## In Response to Your Observations

We recognize that different centers may use varying definitions of cholestasis. In our article, we explained the criteria we applied and discussed how this choice might have influenced our reported incidence. In most cases, our patients’ biochemical markers normalized on their own, without extra treatments or procedures. Direct bilirubin levels were monitored as part of routine exams, meaning the diagnosis did not impact patient comfort, prolong their hospital stay, or require further interventions. At our center, a single definition of cholestasis is used across all conditions. We hope this approach, along with our explanation of diagnostic variability, gives a well-rounded context for interpreting our findings.

Our study focused on postnatal outcomes of cholestasis rather than delivery-related factors, which is why data on the mode of delivery was not included. However, we agree that such data would add depth to the analysis. Additionally, we acknowledge that the severity of HDFN might have influenced the prevalence of preterm deliveries. This is an aspect we plan to incorporate in future studies.

We included all patients diagnosed with HDFN in our analysis, including those with ABO incompatibility. While cases with ABO incompatibility are often milder, cholestasis can still occur. In our cohort, 19 out of 99 patients (19.2%) with HDFN due to ABO incompatibility showed cholestasis. The literature also reports high direct bilirubin levels in such cases, up to 27.7 mg/dL. Including these patients broadens our analysis, highlighting the potential for cholestasis in various types of HDFN, not just Rh incompatibility. This reasoning also guided our decision to include HDFN cases involving the Kidd antigen system.

Since we included all patients diagnosed with HDFN, including those identified after birth, it was not possible to collect bilirubin measurements at the same time for all cases. We recognize this limitation and have discussed it in the article. Retrospective studies have inherent challenges, and we feel excluding certain data, like cord blood bilirubin levels, due to missing results, would reduce the study’s overall value.

Our approach aims to show that cholestasis in HDFN can occur across different patient groups and severities. We have addressed the study’s limitations to provide context for interpreting the results accurately.

Once again, thank you for your thorough review and valuable suggestions, which will guide our future research.
